# Betamethasone gel compared with lidocaine jelly to reduce tracheal tube related postoperative airway symptoms: a randomized controlled trial

**DOI:** 10.1186/s13104-017-2694-6

**Published:** 2017-08-01

**Authors:** Parineeta Thapa, Ravi Ram Shrestha, Sangeeta Shrestha, Gautam Ratna Bajracharya

**Affiliations:** 1 Department of Anesthesiology and Critical Care, B. P. Koirala Institute of Health Sciences, Ghopa, Dharan-18, Sunsari, Nepal; 2 Department of Anesthesiology and Intensive Care, National Academy of Medical Sciences, Bir Hospital, Mahaboudha, Kathmandu, Nepal; 3 Department of Anesthesiology, Paropakar Maternity and Women’s Hospital, Thapathali, Kathmandu, Nepal; 4 Department of Anesthesiology, Intensive Care and Perioperative Medicine, Nepal Medical College (Pvt.) Ltd. Teaching Hospital, Attarkhel, Jorpati, Kathmandu, Nepal

**Keywords:** Betamethasone, Lidocaine, Intubation, endotracheal, Postoperative complications, cough, sore throat, hoarseness

## Abstract

**Background and objectives:**

Post-operative airway symptoms can be troublesome to patients following an uneventful general anesthesia with endotracheal intubation. In this study, we compared the effectiveness of lubricating an endotracheal tube with betamethasone gel or lidocaine jelly with using an unlubricated tube in reducing the incidence and severity of postoperative sore throat, hoarseness and cough.

**Methods:**

This was a prospective, randomized, single-blind comparative study carried out among 120 ASA I and II patients aged 18–65 years undergoing elective surgery under general anesthesia with endotracheal intubation. Patients were randomly divided into three groups of 40 patients each. Endotracheal tube used for patients in group C was unlubricated, while that for group B and group L were lubricated up to 15 cm mark with 2.5 ml of 0.05% betamethasone gel or 2% lidocaine jelly respectively. Incidence and severity of postoperative sore throat, hoarseness and cough were observed at 1, 6 and 24 h following extubation.

**Results:**

At 24 h following extubation, group B had the lowest incidence of postoperative sore throat among the three groups (group B: 12.5% vs group L: 37.5% vs group C: 25%; p = 0.036). Severity of postoperative sore throat at 24 h was less with betamethasone (score 0: 87.5%, 1: 10%) compared with lidocaine (score 0: 62.5%, 1: 37.5%) and control (score 0:75%, 1: 20%) (p = 0.006). Observations at other times and of other variables were comparable.

**Conclusion:**

Wide spread application of 0.05% betamethasone gel to lubricate the endotracheal tube significantly reduces the incidence and severity of sore throat at 24 h of extubation but not of hoarseness or cough.

## Background

Postoperative sore throat (POST), post-extubation cough (PEC) and hoarseness of voice (HOV) are common and well recognized complications following general anesthesia with endotracheal intubation, increasing the postoperative morbidity and distress to the patients. Studies show a wide variation in the incidence of postoperative airway symptoms, ranging from 20% to as high as 100% for POST [1–4], 40–60% for HOV [5, 6] and 30–50% for PEC [4, 5]. Moreover, PEC can also cause adverse effects like hemodynamic instability, raised intracranial and intraocular pressures [7].

Several medications and non-pharmacological methods have shown to reduce the incidence of airway symptoms with variable success rate. These inconsistent results suggest the likelihood of multiple factors associated with these symptoms—like endotracheal tube size, cuff design, duration of surgery, etc. Whatever the cause, the main mechanism postulated is irritation and inflammation of the airway [7, 8]. Although lidocaine is used for airway anesthesia, its use for postoperative airway symptoms have shown variable results [9–13]. Steroids are known for their anti-inflammatory action. Betamethasone gel is a long acting water-soluble glucocorticoid that has been used topically for the treatment of inflammatory lesions of the oral mucosa. It should, thus, provide lubrication as well as anti-inflammatory effect if used for lubrication of the endotracheal tube. So, we hypothesized that wide spread application of betamethasone gel over the endotracheal tube is as effective as the application of lidocaine jelly for reduction of the incidence and severity of POST, HOV and PEC. Previous studies have compared the effect of betamethasone gel with plain lubricant jellies or lidocaine jellies in reducing POST or HOV. But in our study, we wanted to compare the drug with unlubricated tube as well since that is our routine practice. We also tried to see different postoperative airway symptoms like POST, HOV and PEC simultaneously.

## Methods

This prospective, randomized, single blind comparative study was conducted in national academy of medical sciences (NAMS), Bir Hospital, a medical university hospital in Kathmandu, Nepal from April to September 2011 following approval from the institutional review board. After obtaining written informed consent, 120 patients of American society of anesthesiologists-physical status (ASA-PS) I and II, aged 18–65 years of either sex planned for elective surgery under general anesthesia with endotracheal intubation were enrolled for the study. Sample size was calculated based on a previous study, [5] which showed a 100% incidence of POST 24 h postoperatively following intubation with an unlubricated tube. It was calculated that 37 patients in each group would be required to detect a difference of 25% in the incidence with a power of 90% at 5% significant level. A sample size of 40 patients in each group was taken to compensate for any dropout during the study.

Patient’s refusal, surgeries of the oral cavity and pharynx, an anticipated difficult airway, more than two attempts at intubation, use of throat pack, use of nasogastric or orogastric tube perioperatively, patient with upper respiratory tract infection or on steroid, and surgeries lasting longer than 4 h were excluded. We excluded these cases with increased risk of postoperative airway symptoms because of ethical reasons as well as the potential for bias.

The patients enrolled were randomly assigned by lottery method to one of the three equal groups of 40 patients—Group B—where patients were intubated with endotracheal tube lubricated with 0.05% betamethasone gel; group L—endotracheal tube lubricated with 2% lidocaine jelly, and group C—patients intubated with unlubricated tubes. The patients were kept blinded to the group they were assigned. However, the investigator doing the intervention and assessing the outcome were not blinded. The patients were kept nil per oral from the midnight before surgery. Premedication was done the night before surgery with oral diazepam—5 mg for patients weighing below 50 kg and 10 mg for patients 50 kg or above.

On arrival of the patient in the operating room, non-invasive blood pressure, electrocardiograph and pulse oximeter were attached. After securing an intravenous (iv) access premedication was done with iv midazolam 0.04 mg/kg and pethidine 1 mg/kg body weight. Induction of anesthesia was done with a titrated dose of iv propofol. Vecuronium 0.1 mg/kg was given for muscle relaxation. After 3 min of bag and mask ventilation the trachea was intubated with a polyvinyl chloride endotracheal tube (Mallinckrodt™)—7.5 and 7.0 mm internal diameter for male and female patients respectively.

Before intubation, the endotracheal tube was lubricated from the distal end of the cuff to a distance of 15 cm from the tip using 2.5 ml of 0.05% betamethasone dipropionate gel (Betagel™, Micro Labs Limited, Bangalore, India) for patients in group B and with 2.5 ml 2% lidocaine jelly (Xylocaine^®^ 2% jelly, Astra Zeneca Pharma India Limited, Bangalore, India) for patients in group L. The volume of the lubricant was measured using a syringe. The tracheal tubes for the patients in group C were not lubricated.

Immediately after intubation and confirmation of the tube position, the tracheal tube cuff was inflated with room air to keep the intracuff pressure between 18 and 25 mmHg, measured using a tracheal cuff pressure manometer. Anesthesia was maintained with a titrated dose of isoflurane in 100% oxygen. Intravenous bolus of vecuronium 1 mg was repeated intermittently to maintain muscle relaxation. At the end of the surgery, isoflurane was discontinued. Residual neuromuscular blockade was antagonized with neostigmine 0.05 mg/kg and glycopyrrolate 0.01 mg/kg. After the patient was fully awake, the tracheal tube cuff was deflated and the trachea was extubated. Oral suctioning was done only once, just before the tracheal extubation. All the patients were transferred to post anesthesia care unit and supplemental oxygen was provided by facemask for 30 min. Postoperative analgesia was provided with inj paracetamol 15 mg/kg q6h and inj tramadol 50 mg iv q8h. Inj pethidine 1 mg/kg intramuscular was prescribed on as needed basis. The time of tracheal extubation was noted and assessment of the patients for the incidence and severity of POST, HOV and PEC was done at 1, 6 and 24 h after extubation by one of the non-blinded investigators using the four point scales as follows [5, 14].

Postoperative sore throat

- 0 = No sore throat at any time since the operation
- 1 = Minimal sore throat (complains of sore throat only on asking)
- 2 = Moderate sore throat (complains of sore throat on his/her own)
- 3 = Severe sore throat (change of voice or hoarseness, associated with throat pain)

Post-extubation cough

- 0 = No cough at any time since the operation
- 1 = Minimal cough or scratchy throat
- 2 = Moderate cough
- 3 = Severe cough

Hoarseness of voice

- 0 = No evidence of hoarseness at any time since the operation
- 1 = No evidence of hoarseness at the time of interview
- 2 = Hoarseness at the time of interview noted by patient only
- 3 = Hoarseness that is easily noted at the time of interview

Data was collected and recorded as per the proforma and analyzed by means of statistical software—statistical package for the social sciences (SPSS), ver 16 (SPSS for Windows, Version 16.0. Chicago, SPSS Inc.) and appropriate tests—Chi square test for proportions like sex, incidence and severity of postoperative sore throat, cough and hoarseness of voice, and analysis of variance (ANOVA) and independent *t* test for continuous parametric data like age, height and weight. Overall significance level was maintained at p < 0.05.

## Results

A total of 120 patients who met the inclusion criteria were included in the study. None of the patients was excluded after enrollment. Demographic parameters of the patients were comparable among the groups. (Table 1).

**Table 1:** Demography of the patients

	Group B	Group L	Group C	Significance (p value)
Number (n)	40	40	40	
Sex (male/female)	15/25	14/26	13/27	0.89
Age (mean ± SD) in years	41.7 ± 12	37.58 ± 12.8	41.48 ± 13.29	0.26
Weight (mean ± SD) in kg	55.18 ± 10.1	53.33 ± 10.05	55.35 ± 10.44	0.61
ASA I/II	30/10	34/6	29/11	0.36
Duration of surgery in min (mean ± SD)	98.88 ± 36.33	95.88 ± 39.51	107.88 ± 43.42	0.37
Type of surgery				
1. Cholecystectomy	16	11	22	
2. Urosurgery	17	25	14	
3. Others	7	4	4	

**Fig. 1:**
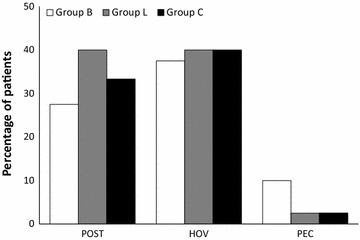
Overall incidence of POST, HOV and PEC

Without the use of any lubricant jelly (group C), the overall incidences of POST, HOV and PEC during the 24-h postoperative period were 33.3, 40 and 2.5% respectively. No significant difference was seen in the incidence of any airway symptom in the 24-h postoperative period among the three groups (p > 0.05) (Fig. 1: overall incidence of POST, HOV and PEC).

**Fig. 2:**
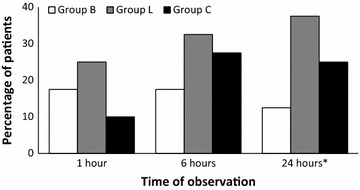
Incidence of POST at different times of observations (*p < 0.05)

When the incidence and severity of POST were considered at different time points, no significant difference was seen at 1 and 6 h post extubation. However, at 24 h following extubation, there was a statistically significant lower incidence of POST in group B compared to the other two groups (group B: 12.5% vs group L: 37.5% vs group C: 25%), p = 0.036. When the groups were compared in pairs, there was a statistically significant difference in the incidence of POST between groups B and L (p = 0.019) with lower incidence of POST in group B. Significant difference in incidence was, however, not found when group C was compared with groups B and L separately. [Figure 2: incidence of POST at different times of observation (*p < 0.05)].

A statistically significant difference in the severity of POST was seen at 24 h following extubation, with lower severity score in group B compared with the other two groups. (p = 0.006). When compared in pairs, there was a significant difference in the severity of sore throat between the groups B and L (p = 0.008) with more severe sore throat in group L compared to group B. When compared between groups B and C or groups C and L however, no significant difference was seen. No statistically significant difference in severity of POST was seen among the groups at other times of observation (Table 2).

**Table 2:** Severity of POST

Time of observation	Severity score for POST	Group B (n = 40)	Group L (n = 40)	Group C (n = 40)	Significance (p value)			
					Among three groups	Between groups B and L	Between groups B and C	Between groups L and C
1 h	0	33	30	36	0.19	0.34	0.51	0.12
	1	6	8	4				
	2	0	2	0				
	3	1	0	0				
6 h	0	33	27	29	0.37	0.09	0.33	0.84
	1	6	11	10				
	2	0	2	1				
	3	1	0	0				
24 h	0	35	25	30	0.006*	0.008*	0.14	0.09
	1	4	15	8				
	2	0	0	2				
	3	1	0	0				

Values given as number of patients

* Significant p value <0.05 (scores compared as ordinal scale using Chi square test)

**Fig. 3:**
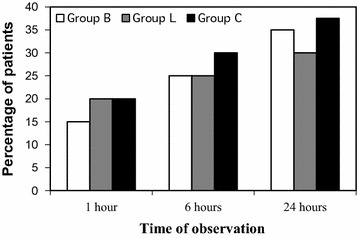
Incidence of HOV at different times of observations

**Fig. 4:**
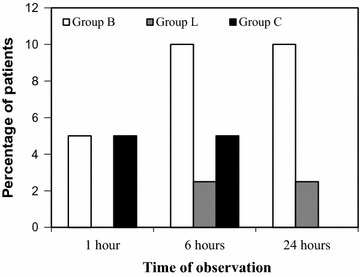
Incidence of PEC at different times of observations

There was a progressive increase in the incidence of HOV with time in all three groups, but no difference was seen among the groups. (Figure 3: incidence of HOV at different times of observations). Most of the patients had mild to moderate hoarseness (Table 3). The incidence and severity of PEC was also comparable at all times of observation. Most of them had only a mild cough. (Figure 4: incidence of PEC at different times of observations) (Table 4).

**Table 3:** Severity of HOV

Time of observation	Severity score for HOV	Group B (n = 40)	Group L (n = 40)	Group C (n = 40)	Significance (p value)			
					Among three groups	Between groups B and L	Between groups B and C	Between groups L and C
1 h	0	34	32	32	0.81	0.55	0.77	1.0
	1	0	2	1				
	2	6	6	7				
	3	0	0	0				
6 h	0	30	30	28	0.24	1.0	0.17	0.30
	1	0	0	3				
	2	8	9	9				
	3	2	1	0				
24 h	0	26	28	25	0.81	0.84	0.73	0.43
	1	3	1	3				
	2	8	8	11				
	3	3	3	1				

Values given as number of patients

**Table 4:** Severity of PEC

Time of observation	Severity score for HOV	Group B (n = 40)	Group L (n = 40)	Group C (n = 40)	Significance (p value)			
					Among three groups	Between groups B and L	Between groups B and C	Between groups L and C
1 h	0	38	40	38	0.54	0.49	1.0	0.47
	1	1	0	2				
	2	1	0	0				
	3	0	0	0				
6 h	0	36	39	38	0.52	0.35	0.67	1.0
	1	3	1	2				
	2	1	0	0				
	3	0	0	0				
24 h	0	36	39	40	0.12	0.35	0.11	1.0
	1	3	1	0				
	2	1	0	0				
	3	0	0	0				

Values given as number of patients

## Discussion

Our study has found a lower incidence of POST—33.3% over 24 h postoperative period compared to most other literature. A study done in Nepal has also found the incidence of POST to be 75, 60 and 50% at 4, 8 and 24 h after extubation, much higher than we found in our study [15]. The incidence of HOV was similar—40%, but the incidence of PEC was very low—2.5%. These variations might be due to various confounding factors like difference in tube sizes, unstandardized cuff pressure, use of nitrous oxide, which is known to increase the cuff volume and pressure or the difference in patient population.

Studies have found a significant reduction of the incidence and severity of POST, HOV and cough with the use of 0.05% betamethasone gel [4, 5, 8, 16]. But we found no significant difference in the incidence over an observation period of 24 h. When we separately considered the incidence and severity at different times of observation, we found a significant difference in POST only at 24 h of observation with lower incidence with the use of betamethasone gel. This may be due to the prolonged anti-inflammatory action of betamethasone gel.

When we compare the incidence over 24 h and at different time points of observation, the incidence of POST was highest with lidocaine jelly at all times, although there was a statistically significant difference only at 24 h. Intergroup comparison showed a significantly high incidence and severity of POST at 24 h with the use of lidocaine compared with betamethasone gel, though the result was not significant when compared with control. This finding is supported by other studies which have shown higher incidence of POST with the use of lidocaine [11, 12]. This is especially prominent with the use of aerosolized lidocaine and is attributed to the additives present in the product [13, 17]. It can also be due to the osmolarity of the jelly since mucosal irritation can depend on the osmolarity of the lubricant [18]. But some other studies have shown a better result with the use of lidocaine [19]. Our significant finding at 24 h may also be due to prolonged action of betamethasone gel while the effect of lidocaine wears off earlier, producing a wider gap in the incidence and severity score at 24 h.

In our study, there was a persistent rise in the incidence of hoarseness of voice with time in all the three groups. This may be because immediately after surgery, patients might have been more concerned about the pain at the operative site than symptoms of the throat. As patients slowly recover from the effects anesthesia, patients would start communicating and notice the hoarseness in their voice. But no significant difference on incidence or severity of HOV was found among the groups.

This finding of higher incidence of HOV with no improvement with the study intervention is unlike the findings by other studies. Most of the studies have found a higher incidence of HOV in control group with significant reduction in incidence and severity with the use of betamethasone. This difference may be due to other factors that we might have overlooked, like environmental factors (colder weather with less maintained room temperature leading to upper respiratory tract infection).

The incidence of PEC was very low in our study—2.5% in the control group with no significant difference among the groups. The severity of PEC was also comparable, but the power of the study may not be adequate to conclude the findings since the incidence of PEC was very low. Other studies have found a much higher incidence of cough than ours.

Our study has various limitations. The study was single blinded. The sample size was powered to the incidence of the events. So, the sample may not be adequate to find the difference in their severity. We had also excluded all the conditions that are high risk for postoperative airway complications. Had we done our study in high-risk patients, for whom these preventive methods should be beneficial, we may have found a better result.

## Conclusion

The use of 0.05% betamethasone gel to lubricate the endotracheal tube prior to intubation is effective in earlier resolution of the symptoms of POST though it does not decrease the incidence of POST, HOV or PEC. It can be useful while intubating a patient with high risk for developing postoperative airway symptoms or patients who are likely to develop complications like cardiovascular instability from symptoms like cough. Use of lidocaine jelly tends to increase the postoperative airway symptoms and should not be used for reducing airway symptoms. Further studies with sample size calculated for HOV, PEC, and severity of symptoms would be needed to improve the power of these findings.

## Abbreviations

ANOVA: analysis of variance
Inj: injection
Iv: intravenous
HOV: hoarseness of voice
PEC: post-extubation cough
POST: postoperative sore throat
SPSS: statistical package for the social sciences
